# eIF2B promotes eIF5 dissociation from eIF2•GDP to facilitate guanine nucleotide exchange for translation initiation

**DOI:** 10.1101/gad.231514.113

**Published:** 2013-12-15

**Authors:** Martin D. Jennings, Yu Zhou, Sarah S. Mohammad-Qureshi, David Bennett, Graham D. Pavitt

**Affiliations:** The Faculty of Life Sciences, The University of Manchester, Manchester M13 9PT, United Kingdom

**Keywords:** protein synthesis initiation, G-protein regulators, GEF, GDF, GDI

## Abstract

Protein synthesis factor eIF2 delivers initiator tRNA to the ribosome. Two proteins regulate its G-protein cycle: eIF5 has both GTPase-accelerating protein (GAP) and GDP dissociation inhibitor (GDI) functions, and eIF2B is the guanine nucleotide exchange factor (GEF). In this study, we used protein–protein interaction and nucleotide exchange assays to monitor the kinetics of eIF2 release from the eIF2•GDP/eIF5 GDI complex and determine the effect of eIF2B on this release. We demonstrate that eIF2B has a second activity as a GDI displacement factor (GDF) that can recruit eIF2 from the eIF2•GDP/eIF5 GDI complex prior to GEF action. We found that GDF function is dependent on the eIF2Bɛ and eIF2Bγ subunits and identified a novel eIF2–eIF2Bγ interaction. Furthermore, GDF and GEF activities are shown to be independent. First, eIF2B GDF is insensitive to eIF2α phosphorylation, unlike GEF. Second, we found that eIF2Bγ mutations known to disrupt *GCN4* translational control significantly impair GDF activity but not GEF function. Our data therefore define an additional step in the protein synthesis initiation pathway that is important for its proper control. We propose a new model to place eIF2B GDF function in the context of efficient eIF2 recycling and its regulation by eIF2 phosphorylation.

## Abstract (precis)

Protein synthesis factor eIF2 delivers initiator tRNA to the ribosome. Two proteins regulate its G-protein cycle: eIF5 has both GTPase-activating protein (GAP) and GDP dissociation inhibitor (GDI) functions, and eIF2B is the guanine nucleotide exchange factor (GEF). Jennings et al. now establish a second activity for eIF2B (as a GDI displacement factor [GDF]) and demonstrate that this function is independent of its GEF activity. This study suggests that eIF2B is a bifunctional protein and defines an additional step in the protein synthesis initiation pathway.

Delivery of initiator methionyl-tRNA (tRNA_i_^Met^) to the ribosome is fundamental to protein synthesis. In eukaryotes, the G protein eIF2 performs this function (Jackson et al. 2010; Hinnebusch and Lorsch 2012). Similar to other G proteins, eIF2 switches between active GTP and inactive GDP-bound states. This eIF2 cycle is critical for continued protein translation, each round driving another initiation event. eIF2 is inactivated by eIF5 GTPase-accelerating protein (GAP) activity during mRNA start codon recognition. eIF2•GDP is then released in complex with eIF5, where eIF5 has a second function as a GDP dissociation inhibitor (GDI) (Jennings and Pavitt 2010a,b). This function limits GDP release, prohibiting spontaneous nucleotide exchange and maintaining eIF2 in its inactive state. Subsequent reactivation of eIF2 is catalyzed by eIF2B, a guanine nucleotide exchange factor (GEF) that stimulates the substitution of GDP for GTP. Under physiological conditions, the affinity of eIF2 for GDP is greater than GTP, so eIF2B GEF activity is critical for exchange efficiency (Panniers et al. 1988; Nika et al. 2000).

eIF2B is particularly complex for a GEF, comprising five subunits (α, β, γ, δ, and ɛ). It represents a critical regulatory component of eukaryotic translation initiation. In response to a number of different stresses, eIF2 is targeted by protein kinases (four in mammalian cells and one in *Saccharomyces cerevisiae*), all of which phosphorylate eIF2 at Ser51 on the α subunit (Pavitt 2005; Jackson et al. 2010; Baird and Wek 2012). The resulting phosphorylated eIF2 (eIF2αP) is a competitive inhibitor for eIF2B, restricting GEF activity and reactivation of eIF2 (Rowlands et al. 1988). eIF2Bαβδ subunits comprise the “regulatory” subcomplex that senses eIF2αP (Pavitt et al. 1998; Pavitt 2005). We provided genetic evidence that eIF5 GDI function is also important for maintaining tight control of translation at this step as well as biochemical evidence that the abundance of the eIF2/eIF5 complex was enhanced when eIF2 phosphorylation was induced by stress (amino acid starvation) (Jennings and Pavitt 2010a).

The ɛ subunit of eIF2B constitutes the GEF catalytic subunit, and its activity is stimulated by the γ subunit (Pavitt et al. 1998), with which it has extensive interactions (Reid et al. 2012). The C-terminal domain (CTD) of eIF2Bɛ is the minimal “catalytic” region and has a W2 HEAT domain structure (Boesen et al. 2004). This region is highly conserved with the CTD of eIF5 (Bieniossek et al. 2006; Wei et al. 2006), and both eIF2Bɛ and eIF5 interact with eIF2 in a similar manner, predominantly interacting with the same site on eIF2, a lysine-rich eIF2β region termed the “K boxes” (Asano et al. 1999; Alone and Dever 2006; Luna et al. 2012). eIF2 and eIF5 are equally abundant, and in yeast, there is a large cellular fraction of the eIF2/eIF5 complex (Singh et al. 2006, 2007). eIF2B is considerably less abundant (∼10-fold). Taken together, these observations raised the question of how eIF2B gains access to eIF2 from eIF2•GDP/eIF5 to promote nucleotide exchange and continued translation.

In some other G-protein systems, an additional factor has been defined that stimulates the release of the G protein from its GDI and is known as a GDI displacement factor (GDF) (Pfeffer 1994; Dirac-Svejstrup et al. 1997; Sivars et al. 2003). The need for a GDF was originally predicted to be necessary for the GTPase Rab9 because of its high GDI affinity. GDIα and prenyl-Rab9 have a *K_d_* ≤23 nM (Shapiro and Pfeffer 1995), which restricts the amount of freely dissociating Rab available for nucleotide exchange. This affinity is identical to that of eIF2•GDP/eIF5 (*K_d_* = 23 ± 9 nM) (Algire et al. 2005), suggesting that an additional factor may be necessary to release eIF2 from eIF5 to allow eIF2B-stimulated exchange. Interestingly, the bacterial pathogen *Legionella pneumophila*-encoded protein SidM was shown to act as both GEF and GDF for the Rab1 GTPase (Machner and Isberg 2007), providing evidence for dual-functioning proteins in bacterially infected cells. We therefore asked whether eIF2B itself might fulfill the GDF role in addition to its function as a GEF. We report here that eIF2B does indeed possess the ability to displace eIF2 from eIF2•GDP/eIF5. We isolated this new activity to the ɛ and γ subunits and identified mutations in eIF2Bγ that impair this function and demonstrate that it is independent of GEF activity. We therefore propose that eIF2B is a bifunctional protein with GDF activity in addition to GEF activity.

## Results

### eIF5 GDI activity antagonizes eIF2B in the absence of eIF2 phosphorylation

**Figure 1.**
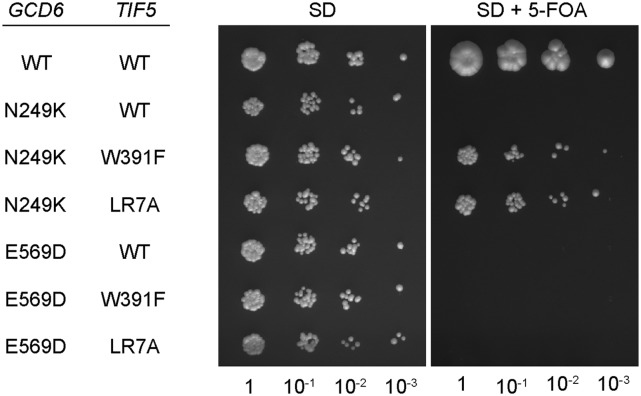
eIF5 GDI mutants restore growth to the lethal eIF2B mutant *gcd6*-N249K. Strains derived from GP5934 *(gcd6*Δ *gcn2*Δ *tif5*Δ) expressing either wild-type (WT) or mutant *tif5 TRP1* plasmids as the sole source of eIF5 and a *GCD6 URA3* plasmid (encoding eIF2Bɛ) were transformed with *LEU2* plasmids expressing either wild-type or mutant *gcd6*, as indicated in the figure. Tenfold serial dilutions of liquid cultures were then plated on SD or SD + 5-fluoro-orotic acid (5-FOA) to inhibit growth of cells that had not lost the covering *GCD6 URA3* plasmid, thereby making the indicated *gcd6* allele the sole source of eIF2Bɛ.

We proposed previously that eIF5 GDI activity is required in vivo in yeast for proper control of protein synthesis in response to stress-induced eIF2 phosphorylation. A mutant yeast strain lacking GDI activity (*tif5*-W391F) was not able to induce *GCN4* translation and grow under conditions of amino acid starvation (Jennings and Pavitt 2010a). Because eIF5 GDI functions biochemically in vitro with unphosphorylated eIF2, we aimed to provide genetic evidence that the GDI function of eIF5 had a role in nonstarved cells. We speculated that eIF5 GDI mutants might reduce or eliminate the requirement for eIF2B GEF activity in vivo. To address this experimentally, we used the two available eIF2Bɛ subunit missense mutants (*gcd6-N249K* and *gcd6-E569D*) that are lethal to yeast cells when expressed as the sole source of eIF2Bɛ with wild-type eIF5 because they reduce GEF activity below the level required for life (Fig. 1, right panel, cf. rows 1, 2, and 5; Mohammad-Qureshi et al. 2007a). We hypothesized that if eIF5 GDI activity antagonizes eIF2B under nonstressed conditions or prevents bypass of eIF2B GEF function, then disrupting GDI function may be able to rescue the lethality of these eIF2B mutants. Single-copy plasmids bearing the eIF2Bɛ N249K or E569D mutations were transformed into eIF2Bɛ and eIF5 double-deleted (*gcd6*Δ *tif5*Δ) yeast cells expressing wild-type *GCD6* on a *URA3* plasmid and either wild-type eIF5 or one of two previously described GDI mutant forms of eIF5 (*tif5*-W391F or *tif5*-LR7A) as the sole source of eIF5 (Fig. 1, left panel). The W391F substitution reduces the affinity of eIF5 for eIF2, and LR7A specifically disrupts GDI activity by weakening the interaction between eIF5 and eIF2γ (Jennings and Pavitt 2010a). As the *gcd6* mutants are recessive, all strains grow normally. Plasmid shuffling was used to permit growth of only cells that have lost the *GCD6 URA3* plasmid so that each *gcd6* mutant became the sole source of eIF2Bɛ. Both eIF5 GDI mutants suppress the lethality of *gcd6*-N249K but not *gcd6*-E569D (Fig. 1, right panel). These results indicate that under nonstress growth conditions, GDI activity limits eIF2 recycling, and loss of eIF5-GDI reduces the requirement for eIF2B GEF function but does not eliminate it. E569D alters a key residue within the catalytic domain and reduces eIF2B activity to background levels in vitro (Mohammad-Qureshi et al. 2007a). In contrast, N249K retains ∼16% GEF activity under similar experimental conditions (Gomez and Pavitt 2000). These data provide further evidence that eIF5 GDI is important in vivo and suggest that it either antagonizes eIF2B GEF function or prevents eIF2B-independent nucleotide exchange. In the experiments described in subsequent sections, we present evidence that support the latter idea and reveal a new function for eIF2B.

### eIF2B possesses GDF activity in addition to GEF activity

**Figure 2.**
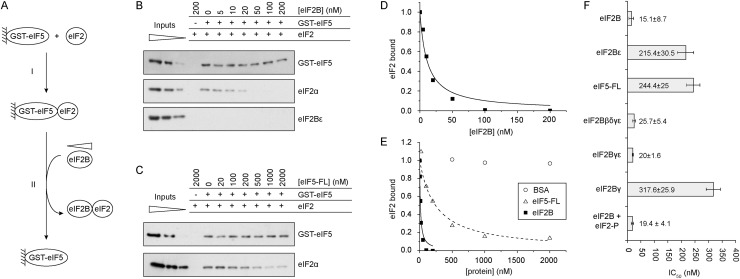
eIF2B acts as a GDF to effectively displace eIF2 from eIF5. (*A*) Scheme of GDF assay. (*B*,*C*) Assay assessing remaining complex formation between GST-eIF5 and eIF2 in the presence of increasing concentrations of either eIF2B (*B*) or eIF5-Flag (*C*). Typical data are shown (*n* > 3). GST-bound proteins were detected with the indicated antibodies. Input samples represent dilutions of proteins used in the assay to assist Western quantification (1.7, 0.83, and 0.33 pmol of eIF2 and eIF2B and 7, 2.9, and 0.7 pmol of eIF5). (*D*,*E*) Signal intensity quantification (Adobe Photoshop) of bound eIF2 from *B*, *C*, and Supplemental Figure S2E. (*F*) Mean IC_50_ ± the standard deviation (*n* > 3) for each protein tested.

As eIF2B and eIF5 share overlapping binding sites on eIF2 (Asano et al. 1999; Alone and Dever 2006) and because eIF5 interacts with a nanomolar *K_d_* to eIF2 (Algire et al. 2005), we set up an in vitro assay to assess how eIF2B accesses eIF2 from the eIF2/eIF5 complex using proteins purified from yeast or bacteria. We used a two-step assay that involved first assembling a glutathione resin-immobilized GST-eIF5/eIF2 complex (Fig. 2A, step I). Next, we challenged this complex with different concentrations of a purified eIF2B complex and monitored dissociation of eIF2 from GST-eIF5 (Fig. 2A, step II). As a control, we replaced eIF2B with increasing concentrations of free purified Flag-tagged eIF5 (eIF5-FL) or an unrelated protein, BSA. Because eIF5 and eIF2B have similar affinities for a shared/overlapping eIF2-binding site (Asano et al. 1999; Algire et al. 2005; Alone and Dever 2006; Gomez and Pavitt 2000; analysis of our published data), we reasoned that if a simple competition model existed, then both eIF2B and a non-GST-tagged version of eIF5 would be able to dissociate eIF2 from GST-eIF5/eIF2 with similar kinetics.

eIF2B dissociated eIF2 from GST-eIF5/eIF2, and we could not detect any stable binding of eIF2B to GST-eIF5 (Fig. 2B). We also found that eIF5-Flag dissociated eIF2 (Fig. 2C), whereas BSA could not (Fig. 2E; Supplemental Fig. S2E). Surprisingly, however, the eIF2B and eIF5-Flag concentrations required for dissociation were considerably different (Fig. 2, cf. B and C). Bound eIF2 was quantified, and dissociation curves were fitted (Fig. 2D,E) to obtain IC_50_ values for factor-promoted dissociation of eIF2 from GST-eIF5 (Fig. 2F). These experiments demonstrated that considerably less eIF2B is required to dissociate eIF2/GST-eIF5 than eIF5-Flag (15.1 ± 8.7 nM vs. 244.4 ± 25 nM) (Fig. 2F). Taken together, these experiments suggest that eIF2B exhibits a specific activity (promoting dissociation of the eIF2/eIF5 complex), whereas eIF5-Flag is limited to competing with GST-eIF5 for interaction with the fraction of eIF2 that has dissociated from the glutathione resin-immobilized GST-eIF5/eIF2 complex. The tight affinity between eIF2 and eIF5 shifts the equilibrium away from release of free eIF2, limiting the ability of free eIF5-Flag to compete for eIF2 interaction, while eIF2B is not similarly inhibited. This is consistent with eIF2B having a novel eIF5 displacement function and that eIF2B is a GDF. In subsequent sections, we further define this activity and its relationship with GEF activity.

### GDF function requires both the eIF2Bɛ and eIF2Bγ subunits

To delineate the regions of eIF2B required for GDF function, various eIF2B subunits and subcomplexes were purified from yeast (Supplemental Fig. S1A). These were used in the same assay (Fig. 2A) as the full eIF2B complex. The ɛ subunit of eIF2B provides the major eIF2 interaction site, with the CTD of eIF2Bɛ representing the minimal site for eIF2 interaction and GEF activity. eIF2Bɛ was unable to displace eIF2/eIF5 with the same efficiency as the full eIF2B complex (Fig. 2F; Supplemental Fig. S2C), suggesting that this subunit alone does not possess GDF ability and that, similar to eIF5, eIF2Bɛ is limited to interacting with freely dissociating eIF2. The eIF2Bɛ CTD and eIF5 CTD share structural and sequence homology and are thought to interact with eIF2 in a comparable way. Consistent with this, eIF2Bɛ and eIF5-Flag have a similar IC_50_ concentration for dissociating eIF2/eIF5 (215.4 ± 30.5 nM and 244.4 ± 25nM, respectively). This result indicates that eIF2B complexes have GDF activity, while eIF2Bɛ alone does not.

The α subunit of eIF2B in yeast is the only nonessential subunit and is required for control of GEF by eIF2 phosphorylation (Pavitt et al. 1998; Pavitt 2005). If GDF activity is an important function of eIF2B, then a four-subunit eIF2B complex lacking the α subunit (eIF2Bβδγɛ) should retain GDF ability. Consistent with this idea and with an eIF2αP-specific role for eIF2Bα, the eIF2Bβδγɛ complex retains GDF ability at a level equivalent to the full eIF2B complex (Fig. 2F; Supplemental Fig. S2A). The eIF2Bγ and eIF2Bɛ subunits constitute the core of eIF2B GEF function, with γ stimulating the GEF activity of ɛ within the “catalytic subcomplex” (Pavitt et al. 1998). We purified the yeast eIF2Bγɛ subcomplex and found that it behaves similarly to the full eIF2B complex in our assay (Fig. 2F; Supplemental Fig. S2B). Because this result contrasted with the behavior of the isolated ɛ subunit, we also purified and assayed the eIF2Bγ subunit alone. eIF2Bγ alone was not sufficient for full GDF function (Fig. 2F; Supplemental Fig. S2D) but was able to dissociate GST-eIF5/eIF2 to an extent similar to that of eIF5-Flag and eIF2Bɛ. This last result suggests that eIF2Bγ alone can bind eIF2 with an affinity similar to that of eIF2Bɛ and eIF5. To assess this directly, we purified (Supplemental Fig. S1B) and then bound all three proteins individually to Flag affinity resin and assessed their ability to bind added eIF2. eIF2Bγ, eIF2Bɛ and eIF5 all bound an equivalent level of eIF2 (Supplemental Fig. S3A). This provides evidence for direct binding between eIF2 and eIF2Bγ

Taken together, these data suggest that the GDF function of eIF2B requires both the γ and ɛ subunits of eIF2B, both of which can make independent contacts with eIF2. This is in contrast to the GEF function, which minimally requires only the eIF2Bɛ CTD (Gomez et al. 2002).

### eIF2B GDF function is required for efficient GEF activity

**Figure 3.**
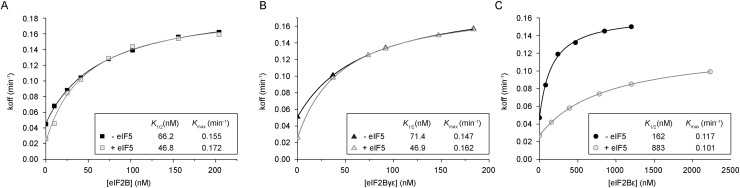
eIF2B GDF activity is required for efficient GEF activity. Rate of [^3^H]GDP dissociation (*K*_off_) from eIF2 was monitored under increasing concentrations of the eIF2B complex (*A*), eIF2Bγɛ (*B*), or eIF2Bɛ (*C*) with GST-eIF5 prebound to eIF2 (gray) or without (black). *K_1/2_* and *K*_max_ values are shown in the *inset* tables, derived from fitting the data to *y* = [(*K*_max_ × *x*)/(*K*_1/2_ + *x*)] + *c*.

To develop a complementary kinetic assay for GDF function, we determined how eIF2B GDF activity affects guanine nucleotide exchange. By assessing the ability of eIF2B to stimulate [^3^H]GDP release from eIF2 (*K*_off_) preincubated with eIF5, we were able to monitor eIF5 GDI and eIF2B GDF and GEF activities in a single coupled assay. As expected, in the absence of eIF5, increasing the concentration of eIF2B stimulated the release of [^3^H]GDP from eIF2 (Fig. 3A; Supplemental Fig. S4). When GST-eIF5 was added to eIF2 in the absence of eIF2B, eIF5 GDI activity stabilized the eIF2•[^3^H]GDP, as we have previously published (Fig. 3A, cf. gray and black symbols at 0 nM eIF2B; Jennings and Pavitt 2010a). However, upon addition of increasing concentrations of eIF2B, this initial stabilization effect by eIF5 GDI becomes nullified as eIF2B concentration is increased. At higher concentrations, eIF2B is able to perform exchange irrespective of the presence of eIF5 (Fig. 3A). This is consistent with a GDF function for eIF2B, meaning that eIF2B is able to readily access eIF2 from preformed eIF2/eIF5 complexes and promote nucleotide exchange. In vivo eIF2 and eIF5 are equimolar, while eIF2B levels are ∼10-fold lower (Singh et al. 2007). In our assay, 42 nM eIF2B mimics this ratio. Above 42 nM eIF2B, the curves with and without eIF5 are superimposable, while when eIF2B is more limiting, competing eIF5 GDI limits exchange.

The catalytic subcomplex of eIF2B (eIF2Bγɛ) exhibited effectively kinetics of nucleotide exchange identical to that of the full eIF2B complex (Fig. 3B; Supplemental Fig. S5A). In accordance with eIF2Bγɛ being sufficient for GDF function (Fig. 2F), this subcomplex was also able to catalyze GEF activity in the presence of eIF5 with kinetics nearly identical to that of the full eIF2B complex (Fig. 3B). The ɛ subunit of eIF2B alone possesses guanine nucleotide exchange activity but requires stimulation by eIF2Bγ for full activity (Supplemental Fig. S5B; Pavitt et al. 1998). In our steady-state GDF-binding assay, this subunit could not efficiently displace eIF2 from GST-eIF5/eIF2 (Fig. 2F). Accordingly, the presence of eIF5 antagonized the ability of eIF2Bɛ to promote the release of [^3^H]GDP from eIF2 in the kinetic assay (Fig. 3C). These observations fit a model where, on its own, eIF2Bɛ is not able to actively displace eIF2 from eIF2•GDP/eIF5 and thus is limited to interacting with freely dissociated eIF2. This restricts the amount of eIF2•[^3^H]GDP available for nucleotide exchange, which increases the observed *K*_1/2_ (Fig. 3C). Taken together, these data indicate that the GDF function of eIF2B is required for efficient eIF2B-catalyzed guanine nucleotide exchange when eIF2/eIF5 is the substrate—the GDF function allowing removal of eIF5 to then permit GEF activity.

### The GDF function of eIF2B is unaffected by eIF2α phosphorylation

**Figure 4.**
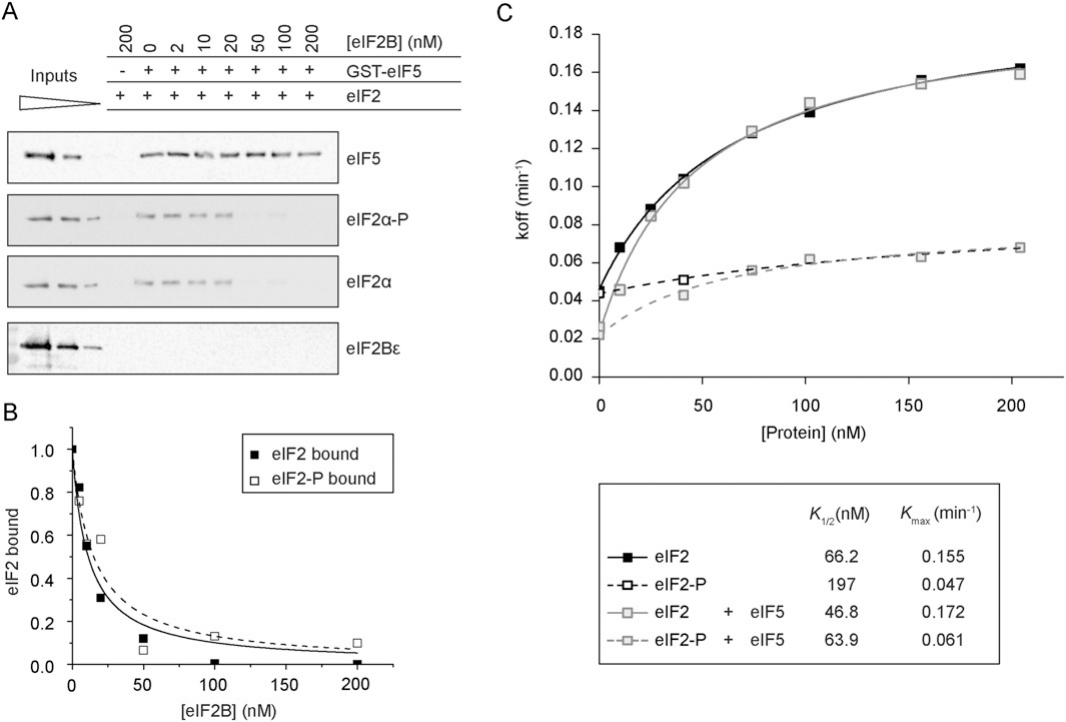
eIF2B GDF is independent of eIF2α phosphorylation. (*A*) The GDF assay depicted in Figure 2A using phosphorylated eIF2, GST-eIF5, and eIF2B. Inputs represent 1.7, 0.83, and 0.33 pmol eIF2 and eIF2B and 7, 2.9, and 0.7 pmol of eIF5. (*B*) Quantification of *A* (open symbols). Unphosphorylated eIF2 quantification is shown for comparison (filled symbols), as shown in Figure 2D. (*C*) The *top* panel shows the rate of [^3^H]GDP dissociation (*K*_off_) from eIF2αP (broken lines) or eIF2 (solid lines) with increasing concentrations of eIF2B either with (gray symbols) or without GST-eIF5 (black symbols). The *bottom* panel shows the legend with quantified *K*_1/2_ and *K*_max_ values.

As outlined above, phosphorylation of eIF2α at Ser51 is one conserved mechanism of translational control in all eukaryotes studied. eIF2 phosphorylation inhibits eIF2B GEF activity by binding in a non-GEF-competent manner involving the regulatory subcomplex of eIF2B (αβδ), increasing the affinity of eIF2αP for eIF2B. Because eIF5 GDI activity modulates the response to eIF2 phosphorylation (Jennings and Pavitt 2010a) and because the abundance of the eIF2/eIF5 complex also increases in cells starved for amino acids, where eIF2αP levels are high, we wished to assess the impact of eIF2αP on the GDF and GEF functions of eIF2B. Our eIF2 was purified from a *gcn2*Δ strain and so was not phosphorylated at Ser51. We purified the eIF2α kinase PKR using a yeast expression system (Krishnamoorthy et al. 2001) and then phosphorylated eIF2 to saturation and used this eIF2 in our assays. We found that phosphorylation of eIF2 had no significant impact on eIF2B GDF in the equilibrium binding assay (Fig. 4A,B), generating IC_50_ values not significantly different from reactions with nonphosphorylated eIF2 (eIF2 = 15.1 ± 8.7; eIF2α*P* = 19.4 ± 4.1) (Fig. 2F).

Phosphorylated eIF2 (Supplemental Fig. S5F) was also used in GEF assays with eIF2B and coupled GDI–GDF–GEF assays. As expected, phosphorylation of eIF2 inhibited eIF2B-catalyzed [^3^H]GDP release (Fig. 4C; Supplemental Fig. S5C). We also found that eIF2αP•[^3^H]GDP is stabilized by eIF5 GDI in the absence of eIF2B to an extent similar to nonphosphorylated eIF2 (Fig. 4C, cf. GDP dissociation ± eIF5 at 0 nM eIF2B; see also Supplemental Fig. S5D). We interpret this result as indicating that the affinity of eIF5 for eIF2 and the resulting GDP stabilization are unaffected by eIF2 phosphorylation. Upon addition of eIF2B to eIF2αP/eIF5, there is a modest rise in [^3^H]GDP release when limiting concentrations of eIF2B are used (Supplemental Fig. S5D). As eIF2B concentration is increased, [^3^H]GDP release reaches the level seen with eIF2αP alone (without eIF5). Thus, eIF2B GEF function with eIF2αP/eIF5 as a substrate is inhibited relative to GEF activity with eIF2/eIF5 (Fig. 4C; Supplemental Fig. S5E). From the data shown in Figure 4 and Supplemental Figure S5, C–F, we propose that the small increase in [^3^H]GDP release seen when eIF2B is added to eIF2αP/eIF5 is likely not due to eIF2B GEF exchange but instead represents displacement of eIF5 by eIF2B GDF (Fig. 4A) and accompanying loss of eIF5 GDI activity (Supplemental Fig. S5D). This explanation fits with the observation that at higher concentrations of eIF2B, the level of [^3^H]GDP release from eIF2αP is similar in the presence or absence of eIF5 (Fig. 4C). The stabilization effect of eIF5 GDI on [^3^H]GDP binding is negated by addition of eIF2B. These data support the idea that GDF and GEF are separate functions of eIF2B, as the GEF function is inhibited significantly by eIF2αP, but GDF activity is not affected.

### Genetic characterization of eIF2Bγ mutants

Our studies imply that eIF2Bγ has a significant role in GDF function. Mutations in *GCD1*, the yeast gene encoding eIF2Bγ, were described previously that impair translational control of *GCN4* mRNA. For example, *gcd1-101* was initially described in 1975 (then called *tra3-1*) (Wolfner et al. 1975) and has been used extensively by the Hinnebusch laboratory (Mueller et al. 1988; Grant et al. 1994) as a model Gcd^−^ allele for studies deciphering the mechanism of *GCN4* control. A separate genetic screen for Gcd^−^ mutants isolated six alleles, termed *gcd1-501* to *gcd1-506* (Harashima and Hinnebusch 1986). The molecular defect underlying each mutation was not identified in any of these published studies, but all impair yeast growth rate, implying that they alter an essential eIF2B function. We therefore obtained strains bearing each of the seven mutations from the Hinnebusch laboratory and sequenced the complete *GCD1* ORF and adjacent genomic DNA from each strain and uncovered molecular defects in each (Supplemental Fig. S6A). A single nucleotide change in the *gcd1-101* allele results in a change from Gly12 to valine (G12V). Three of the *gcd1-500* series alleles also contained single missense mutations, while a fourth altered two amino acids. The remaining alleles revealed no changes to the coding sequence but altered adjacent bases in the promoter region, assigned recently as bases critical for binding the Reb1p transcription factor (Rhee and Pugh 2011). We presume that reduced Reb1p binding would lower eIF2Bγ protein levels but did not directly assess this. eIF2Bγ shares sequence and predicted structural similarity with a family of phospho-hexose sugar nucleotide pyrophosphorylases. eIF2Bγ is therefore predicted to have an N-terminal globular pyrophosphorylase-like domain (PLD) and a CTD that folds into a left-handed β helix (LβH) (Reid et al. 2012). The mutations that we identified are found throughout eIF2Bγ including substitutions in both the PLD and LβH domains (Supplemental Fig. S6C, top panel).

It was previously reported that the slow-growth phenotype of a *gcd1-502* (L480Q) strain is suppressed by overexpression of the three subunits of eIF2 (Dever et al. 1995). Elevated eIF2 protein levels should increase the proportion of eIF2•GDP not bound by eIF5, thereby freeing eIF2 for nucleotide exchange. We thought that the growth suppression phenotype was consistent with the idea that *gcd1-502* has a GDF defect, conferring slow growth, which is then rescued by excess eIF2. We therefore extended the phenotypic analysis to all seven *gcd1* alleles and found that only *gcd1-101* (G12V) and *gcd1-502* (L480Q) were suppressed by excess eIF2 (Supplemental Fig. S6A,B). We therefore decided to focus our attention on these two mutants only. Overexpression of eIF5 enhances complex formation between eIF2 and eIF5 and exacerbates the growth defects of certain eIF2B mutants (Singh et al. 2006). Excess eIF5 also exacerbates growth of both *gcd1* mutants (Supplemental Fig. S6D). In contrast, growth is partially rescued when the eIF5-W391F mutant with a defect in GDI function is overexpressed (Jennings and Pavitt 2010a). Taken together, the genetic observations suggest that G12V and L480Q are candidate mutations defective in GDF function.

### eIF2Bγ mutants impair GDF function

Site-directed mutagenesis was used to introduce the G12V and L480Q mutations into our co-overexpression and purification strains. Mutant proteins were purified and found to have no eIF2B subunit composition/integrity defects (Supplemental Fig. S1C). We assessed interaction with eIF2 in a Flag affinity capture experiment and found no significant eIF2-binding defects with either purified mutant eIF2B complex (Supplemental Fig. S3B). Together, these experiments suggest that the selected mutants do not have a significant defect in eIF2B structure and eIF2 interactions.

**Figure 5.**
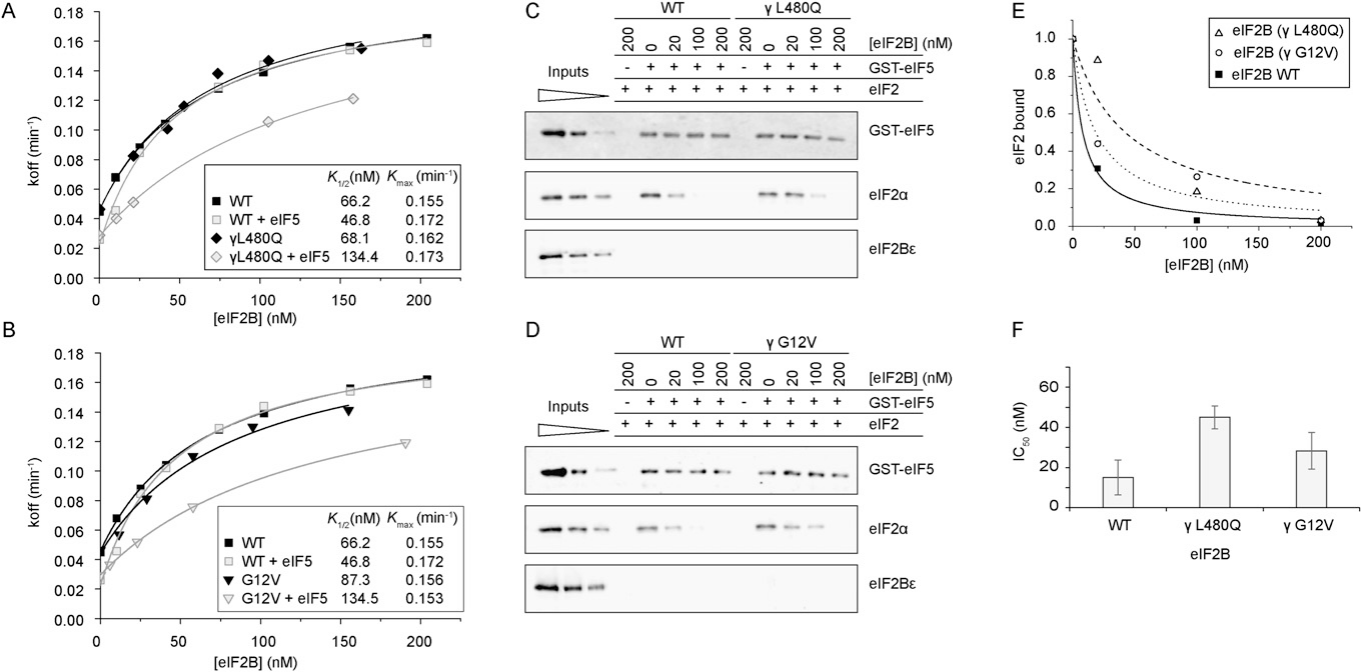
eIF2Bγ mutants have a GDF activity defect. (*A*,*B*) GEF and coupled GDI–GDF–GEF assays of eIF2Bγ mutants L480Q (*A*) and G12V (*B*) are shown, as per Figure 3. (*C*,*D*) GDF assay of eIF2 displacement from eIF5 for the same mutants. (*E*,*F*) Quantification of GDF assays; mean IC_50_ ± standard deviation (*n* = 3).

We next examined the GEF activity of each mutant. eIF2B-γL480Q has no significant effect on the kinetics of nucleotide release (Fig. 5A), while eIF2B-γG12V modestly increased *K_1/2_* but not *K*_max_. At physiological protein ratios, eIF2B-γG12V retains 85% of wild-type activity in this assay (Fig. 5B). When adding eIF5 to our coupled GDI–GDF–GEF assay to assess GDF function, both mutants revealed significantly altered kinetics of nucleotide exchange, increasing *K_1/2_* from 46 to 134 nM (Fig. 5A,B). These data are consistent with each mutant having a significant defect in releasing eIF5 from eIF2 prior to nucleotide exchange (GDF function). We also assessed GDF directly in our previously described equilibrium binding assay (Fig. 2A) and confirmed that both mutants exhibit impaired ability to release eIF5 from eIF2 (Fig. 5C–F). Thus, in summary, we provide here evidence for the importance of GDF in vivo because two mutants in eIF2Bγ that were isolated as regulators of *GCN4* translational control have a major defect in eIF2B GDF activity but only a minor or no defect in GEF function.

## Discussion

### eIF2B is a dual-function protein with GDF and GEF activities

**Figure 6.**
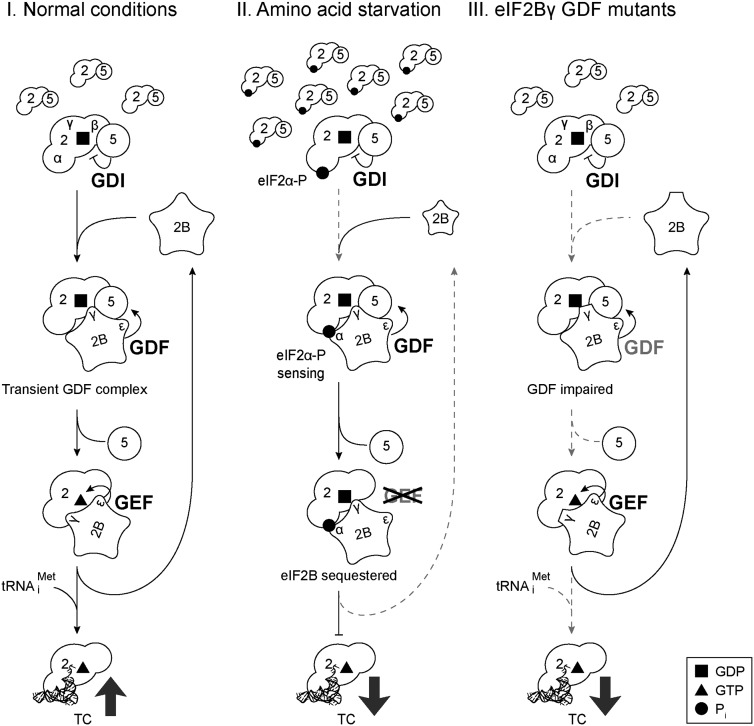
Revised model for eIF2 ternary complex formation and its inhibition by phosphorylation of eIF2. Model showing proposed paths for eIF2 (2, two-lobed ovals), eIF5 (5, open circles), and eIF2B (2B, open five-pointed stars) integrating eIF5 GDI and eIF2B GDF and GEF activities in the recycling of eIF2 (part I), its inhibition by eIF2αP (part II), and the affect of eIF2Bγ GDF mutations (part III). Dashed gray arrows represent steps that limit eIF2 recycling.

eIF2 and eIF5 interact with high affinity (Algire et al. 2005) and have been shown to exist as an abundant cellular fraction (Singh et al. 2006, 2007). The nature and high affinity of such a G-protein•GDP/GAP complex is atypical of most G-protein systems studied. We showed previously that yeast eIF5 possesses GDI activity, stabilizing eIF2•GDP (Jennings and Pavitt 2010a) and providing functionality to the eIF2•GDP/eIF5 complex. Other studies have revealed that the structures of the eIF5 and eIF2Bɛ CTDs are highly similar (Boesen et al. 2004; Bieniossek et al. 2006) and that equivalent residues are important for interacting with eIF2; e.g., W699 in eIF2Bɛ and W391 in eIF5 (Asano et al. 1999; Alone and Dever 2006; Mohammad-Qureshi et al. 2007a; Jennings and Pavitt 2010a). These and other data strongly suggest antagonism between eIF2B and eIF5 for interaction with eIF2 (Singh et al. 2006). eIF2 has a much higher affinity for GDP than GTP (Erickson and Hannig 1996), raising an interesting dilemma about how the essential step of eIF2B-catalyzed guanine nucleotide exchange occurs efficiently; i.e., how eIF2B accesses eIF2 when eIF2 is bound to eIF5. Here we showed by biochemical and genetic approaches that eIF2B possesses GDF activity in addition to GEF activity (Figs. 2–6).

As eIF2B can effectively displace eIF5, the evidence that disruption of eIF2•GDP/eIF5 can rescue growth of a lethal eIF2B mutant (Fig. 1) suggests that the eIF2•GDP/eIF5 complex predominately prevents spontaneous GDP/GTP exchange (GDI) rather than antagonizes eIF2B GEF activity. The eIF5 GDI mutants permit some bypass of the defective eIF2B GEF function. The fact that disruption of GDI activity does not fully recover growth in the *gcd6*-N249K mutant and does not rescue viability in *gcd6*-E569D mutant cells indicates that the level of spontaneous exchange alone is not sufficient for efficient translation initiation in the absence of other factors. This is in line with previous observations. For example, it is possible to bypass the requirement for eIF2B in yeast by co-overexpression of eIF2 and tRNA_i_^Met^ (Erickson et al. 2001; Gomez et al. 2002). In the eIF2B bypass strains, poor growth was rescued by a mutation in eIF2γ that weakened eIF2γ's affinity for GDP (Erickson et al. 2001).

In an effort to provide experimental evidence that eIF2B GDF and GEF functions are distinct, we first attempted to purify a “GEF-dead” E569 mutant of eIF2B in our overexpression system. This was unsuccessful, probably because mutant eIF2Bɛ expression was toxic. Instead, we examined the effect of eIF2α phosphorylation on wild-type eIF2B GEF and GDF functions. Our results (Fig. 4) clearly demonstrate that eIF2B GEF function is inhibited by eIF2αP, while GDF activity is retained. In contrast, our biochemical analysis of eIF2Bγ mutants revealed no (L480Q) or only slight (G12V) reduction in GEF activity, while both mutants have significant GDF defects increasing *K_1/2_* from 47 nM to 134 nM for GDP release (Fig. 5). These experiments provide strong complementary evidence that the two activities are distinct functions of eIF2B.

### eIF2B GDF and human disease

Mutations in human eIF2B cause fatal eIF2B-related disorders, also known as leukoencephalopathy with vanishing white matter or childhood ataxia with CNS hypomyelination (van der Knaap et al. 2002; Pavitt and Proud 2009). The mutations affect any eIF2B subunit, and initial reports found that various mutations reduced eIF2B GEF activity (Fogli et al. 2004; Li et al. 2004; Richardson et al. 2004). However, more recent studies have identified eIF2B mutations that do not appear to significantly reduce eIF2B GEF function, despite some being responsible for severe forms of disease (Horzinski et al. 2009; Liu et al. 2011). Four *EIF2B3* (human eIF2Bγ) mutants have been analyzed biochemically using purified proteins: L27Q, Q136P, R225Q, and H341Q (Liu et al. 2011; Matsukawa et al. 2011). None had obvious defects in eIF2B complex formation. L27Q was reported to have the severest defect in GEF activity (∼65% of wild type) but was associated with the mildest adult-onset form of disease (Matsukawa et al. 2011). However, mutations associated with more severe disease retained more GEF activity (e.g., R225Q with ∼95%) (Liu et al. 2011). These and other similar data led the investigators to conclude that eIF2B may have other functions. Interestingly, the human mutation G11V is an amino acid substitution equivalent to our γG12V allele; human eIF2Bγ has three adjacent glycine residues at positions 11–13, where yeast eIF2Bγ has a single glycine at position 12. The human mutation situated closest to our γL480Q mutation is I375S (Supplemental Fig. S6C). Although they are not equivalent in sequence alignments, three-dimensional structural homology models suggest that yeast L480 and human I375 are situated close to each other within the center of the LβH domain. In common with analyses of the human mutations, we found no defects in yeast eIF2B complex formation, gross eIF2 interaction defects, or GEF dysfunction for the two mutants studied (Fig. 5; Supplemental Figs. S1C, S3B). As these mutations do impair GDF and impact on yeast cell growth (Fig. 5; Supplemental Fig. S6) and *GCN4*-regulated translational control (Harashima and Hinnebusch 1986; Harashima et al. 1987), our data demonstrate that GDF function is critical for both normal protein synthesis and eIF2B-mediated translational control and therefore has the potential to be critical in the context of eIF2B related disorders.

### Comparison of eIF2B GDF with other GDFs

Proteins with GDF activity have also been described for some small GTPases. A dedicated GDF that does not possess GEF function, called Yip3/Pra1, has been described for the Ras-like GTPase Rab9 (Dirac-Svejstrup et al. 1997). Rab9 binds GDIα, and Yip3/Pra1 releases GDIα (Sivars et al. 2003) so that Rab9 can be activated by separate GEFs (Yoshimura et al. 2010). As different proteins provide GDF and GEF functions, the findings clearly show that the two activities are separable. This agrees with our own analysis, as eIF2B GDF is largely unaffected by eIF2αP, while eIF2B GEF is inhibited (Fig. 4).

To our knowledge, the only dual-function GDF/GEF protein described also functions with Rab GTPases. The intracellular human pathogen *L. pneumophila* expresses proteins that modify the function of host processes. The bacterial protein SidM (also called DrrA) is translocated into the host cytoplasm and possesses dual GEF and GDF activity toward host G protein Rab1 (Machner and Isberg 2007). Structural and biochemical studies have shown that the same surface of SidM/DrrA is involved in both displacement of GDI from Rab1 and nucleotide exchange functions, which differs from our own observations for eIF2B. The distinction between SidM/DrrA and other Rab GEFs is in its ability to both release RabGDI and bind with high affinity to Rab1•GDP, retaining GDP in the active site (Suh et al. 2010). We also observed GDF without GEF for the interaction between eIF2B and eIF2αP/eIF5 (as discussed below). The proposed model for SidM/DrrA GDF involves formation of a transient three-way complex between Rab1•GDP/GDI and SidM/DrrA, although this complex eluded biochemical detection (Suh et al. 2010).

### Integrating eIF2B GDF activity into models for eIF2 recycling and its control

Our data favor a model for eIF2 recycling (depicted in Fig. 6, part I) in which eIF2•GDP is released from initiating ribosomes in complex with eIF5. eIF5 binding to eIF2 limits spontaneous GDP release (GDI activity) and accumulates as a pool of eIF2/eIF5 (Singh et al. 2006; Jennings and Pavitt 2010a). Next, eIF2B interacts with this complex, presumably at a site that is nonoverlapping with the eIF5/eIF2 interface to form a transient three-way eIF2B/eIF2/eIF5 complex prior to displacing eIF5 (GDF function) to form an eIF2/eIF2B complex competent for nucleotide exchange (GEF). Because we identified a stable interaction between eIF2Bγ alone and eIF2 (Supplemental Fig. S3A) and our mutant data indicate a significant role for eIF2Bγ (Fig. 5), here we depict eIF2Bγ making initial contact with eIF2. We postulate that the eIF5/eIF2/eIF2B complex is transient as we could not detect a stable interaction of eIF2B with GST-eIF5 prior to eIF5 displacement (Fig. 2B; Supplemental Fig. S2), although such a complex has been observed previously in extracts from yeast cells overexpressing eIF5 (Singh et al. 2006). That study suggested that excess eIF5 stabilized a transient intermediate such as we propose here. A similar complex was also proposed for the GDF action of SidM (Suh et al. 2010).

Phosphorylation of eIF2α has long been known to regulate both global and gene-specific translation (Jackson et al. 2010). eIF2αP binds eIF2B to form a stable complex that does not promote nucleotide exchange (Fig. 4; Rowlands et al. 1988; Pavitt et al. 1998). The experiments presented here show that eIF2B GDF activity is independent of eIF2α phosphorylation (Fig. 4A,B). This is consistent with our findings that efficient GDF activity requires only eIF2Bγ and eIF2Bɛ. In contrast, it is well established that eIF2αP sensing is mediated by the eIF2Bαβδ regulatory subcomplex (Yang and Hinnebusch 1996; Pavitt et al. 1997; Krishnamoorthy et al. 2001). Thus, when eIF2α is phosphorylated, eIF5 can be displaced by free eIF2B to form inhibitory eIF2αP•GDP/eIF2B complexes (Fig. 6, part II). However, in vivo, as concentrations of eIF2αP/eIF2B rise, free eIF2B becomes limited in availability. This effectively reduces eIF2B GDF and consequently increases levels of the eIF2/eIF5 GDI complex, as observed (Jennings and Pavitt 2010a). In this model, eIF5 is acting as a buffer in the eIF2 regulatory pathway, where it absorbs the backlog of eIF2 released from initiating ribosomes, and its GDI prevents any spontaneous nucleotide exchange, ensuring tight regulatory control (Fig. 6, part II). Consistent with this model, mutants with a defect in GDF function at a different point to restrict access of eIF2B to eIF2 (Fig. 6, part III), reducing GDF activity, which effectively lowers nucleotide exchange activity and ternary complex levels. This model explains the slow growth and Gcd^−^ phenotypes, which are indistinguishable from mutants that lower GEF activity directly.

In summary, the model shown in Figure 6 depicts eIF2 recycling following its release from the ribosome. It integrates eIF5 GDI and eIF2B GDF and GEF functions to generate eIF2•GTP•Met-tRNA_i_^Met^ ternary complexes and the inhibition of eIF2B GEF by eIF2αP. This model predicts that eIF2 is almost always bound by other factors in vivo and is handed over from one to the next during successive translation initiation cycle steps.

## Materials and methods

### Yeast genetics

Yeast strains were grown in standard medium as described (Amberg et al. 2005). Plasmid transformations used the lithium acetate method (Gietz and Woods 2006), and plasmid shuffling used unselected segregation or 5-fluoro-orotic acid (5-FOA) as described (Amberg et al. 2005). *TIF5*-Flag was subcloned as a HindIII–EcoRI fragment from YEpTIF5-Flag into the *TRP1* vector YCplac22 (Gietz and Sugino 1988), creating pAV2178. A similar strategy was used to create W391F and LR7A mutant variants pAV2179 and pAV2180 (Supplemental Table S1; Jennings and Pavitt 2010a). Site-directed mutagenesis (QuikChange, Agilent Technologies) using primers listed in Supplemental Table S2 was used to introduce G12V and L480Q mutations into the high-copy *GCD1/GCD6* plasmid pAV1413 [*GCD1*-Flag_2_-His_6_ *GCD6 LEU2* 2 μm], giving pAV2343 and pAV2344 (Supplemental Table S1). The *gcd6Δ tif5Δ* double-shuffle strain GP5934 (*MAT***a** *ura3-52 leu2-3 leu2-112 trp1Δ63 gcn2Δ tif5Δ gcd6Δ*∷KanMX4 [*TIF5 URA3*] pAV1369 [*GCD6 TRP1*]) was created by transformation of the *tif5*Δ strain H2786 (Asano et al. 1999) with pAV1369 and targeted disruption of the genomic copy of *GCD6* with a *gcd6Δ*∷KanMX4 PCR fragment from Euroscarf strain Y23570 (BY4743 *gcd6*Δ∷KanMX4/*GCD6*). A series of plasmid-shuffling experiments was then conducted that ultimately introduced plasmids with specific *tif5* alleles on *TRP1* plasmids (pAV2178 [*TIF5*], pAV2179 [*tif5*-W391F], and pAV2180 [*tif5*-LR7A]) along with pAV1272 [*GCD6 URA3*] and the dead mutant *gcd6 LEU2* plasmids (pAV1590 [*gcd6*-N249K] and pAV2132 [*gcd6*-E569D]). 5-FOA was used to identify strains growing without pAV1272, as shown in Figure 1. Plasmids were rescued from the mutant strains that grew on 5-FOA and sequenced to confirm the presence of the original *gcd6* and *tif5* mutations. *gcd1-101* and *gcd1-501–gcd1-506* strains listed in Supplemental Figure S6 were obtained from Alan Hinnebusch (National Institutes of Health). Strains were transformed with *URA3*-marked plasmids listed in Supplemental Table S1 bearing *GCD1*, all three genes encoding eIF2 subunits, high-copy eIF5, the *W391F* allele, or no insert, as indicated in the legend. Genomic DNA from wild-type and mutant strains was isolated using standard methods for PCR, and sequencing of *gcd1* alleles was performed using the primers indicated in Supplemental Table S2. Site-directed mutagenesis (QuikChange, Agilent Technologies) was used to introduce G12V and L480Q mutations into the high-copy plasmid pAV1413 [*GCD1*-Flag_2_-His_6_ *GCD6 LEU2* 2 μm]. Plasmids are listed in Supplemental Table S1, and primer sequences are given in Supplemental Table S2. Strains used for protein purification are described below.

### Protein purification

eIF2 was purified using strain GP3511 as described previously (Pavitt et al. 1998). GST-eIF5 was purified from *Escherichia coli*, and eIF5-Flag was purified from yeast as described previously (Jennings and Pavitt 2010a). Flag-tagged PKR was purified from a yeast strain resistant to the toxic effects of PKR expression (GP3299) and harboring plasmid pAV1412 as described previously (Krishnamoorthy et al. 2001). eIF2B complexes, subcomplexes, and subunits were all purified from yeast as described (Mohammad-Qureshi et al. 2007b) using protease-deficient strains bearing high-copy-number plasmids overexpressing the required factor genes from 2-μm plasmids BJ1995[GP3583] (*MAT*α *prb1-1122 pep4-3 leu2 trp1 ura3-52 gal2*) or GP4597(*MAT*α *trp1Δ63 ura3-52 leu2-3 leu2-112 GAL2 gcn2Δ pep4∷LEU2*). Plasmids used are described in Supplemental Table S1. Proteins were eluted from Flag M2 affinity resin (Sigma) with 3xFlag peptide, dialyzed into storage buffer (30 mM HEPES at pH 7.4, 100 mM KCl, 10% glycerol, 0.1 mM MgCl_2_, 0.1% Triton X-100, 5 mM DTT), and stored at −80°C.

### Phosphorylation of eIF2 in vitro

Purified eIF2 was phosphorylated using purified PKR. Typically, 5 μg of eIF2 was incubated with 0.3 μL of PKR, 0.1 mM ATP, and 5 mM NaF for 15 min at room temperature.

### SDS-PAGE and immunoblotting

SDS-PAGE and immunoblotting were performed as described previously (Jennings and Pavitt 2010a) using specific antibodies for eIF5, eIF2α, eIF2Bɛ, and eIF2Bγ. Horseradish peroxidase-conjugated secondary antibodies and chemiluminescent detection were performed as per the manufacturer's instructions (Perkin Elmer). In addition, quantitative IR Western blot detection was performed using IRDye 800CW goat anti-rabbit IgG or IRDye 680RD goat anti-mouse IgG followed by detection with an Odyssey Fc imaging system (Li-Cor).

### GDF assay

One microgram of eIF2 or eIF2α-P and 1 μg of eIF5 were incubated with 20 μL of glutathione sepharose beads in 100 μL of binding buffer (30 mM HEPES, 100 mM KCl, 100 μM GDP, 5 mM DTT, 1 mM NaF, 2.5 mM MgCl_2_, 0.05% Triton X-100) for 2 h at 4°C with mixing. Binding was done at 4°C to maintain protein stability. This was then washed twice with 100 μL of binding buffer. Various concentrations of Flag-eIF2B complexes/eIF5-Flag or BSA were then added in 100 μL of binding buffer for a further 2 h at 4°C with mixing. Beads were then washed three times with 100 μL of binding buffer before being boiled in 20 μL of Laemmli sample buffer and analyzed by SDS-PAGE and immunoblotting. Quantified binding was fitted to *y* = 1 − [*x*/(*x* + IC_50_)] to calculate an IC_50_ value.

### [^3^H]GDP filter-binding GEF assay

eIF2•[^3^H]GDP binary complexes were formed in 75-mm × 12-mm soda-lime glass tubes (Fisher) using 42 pmol of eIF2 and 75 pmol of [^3^H]GDP in assay buffer (30 mM HEPES at pH 7.5, 100 mM KCl, 0.1 mM EDTA, 1 mM DTT, 2 mg mL^−1^ BSA). Complex formation was carried out for 10 min at room temperature before stabilization by addition of MgCl_2_ to 3 mM and incubation for a further 2 min at room temperature. Purified eIF5 (42 pmol) or sample buffer was then bound to eIF2•[^3^H]GDP by incubation for 30 min at 10°C. Temperature and [MgCl_2_] conditions used were optimized to permit experimental measurement while allowing for further stabilization or destabilization of GDP dissociation. Dissociation and nucleotide exchange were initiated by addition of a >100-fold excess of unlabeled GDP (20 nmol) in addition to either eIF2B (1–20.4 pmol for wild type) or sample buffer. Samples (12 μL) were taken immediately (*t* = 0) and at 2, 4, 6, 8, and 10 min. At each time point, samples were added to 2.5 mL of ice-cold Stop buffer (30 mM HEPES at pH 7.5, 100 mM KCl, 0.1 mM EDTA, 5 mM MgCl_2_), filtered through Whatman 0.45-μm 25-mm cellulose nitrate filters using a Millipore vacuum manifold, and then washed twice with 2.5 mL of ice-cold Stop buffer. Filters were dried at 65°C and then counted by liquid scintillation in Ultima Gold F (Perkin Elmer). Experimental data were fitted to exponential dissociation curves to obtain the dissociation rate constant (*K*_off_).

### Flag affinity binding with purified proteins.

Flag-tagged proteins (30 pmol) and eIF2 (40 pmol) were incubated with 30 μL of Flag M2 affinity resin (Sigma) in 500 μL of binding buffer (30 mM HEPES, 100 mM KCl, 100 μM GDP, 5mM DTT, 1mM NaF, 2.5 mM MgCl_2_, 0.05% Triton X-100) for 2 h. The beads were then washed twice with 1 mL of binding buffer before boiling in 40 μL of Laemmli sample buffer and analysis by SDS-PAGE and immunoblotting.
